# Diabetes distress is associated with adverse pregnancy outcomes in women with gestational diabetes: a prospective cohort study

**DOI:** 10.1186/s12884-019-2376-6

**Published:** 2019-07-03

**Authors:** Charlotte B. Schmidt, Ilse Voorhorst, Vital H. W. van de Gaar, Anne Keukens, Bert Jan Potter van Loon, Frank J. Snoek, Adriaan Honig

**Affiliations:** 1 Department of Psychiatry, OLVG, Amsterdam, the Netherlands; 2 Amsterdam Public Health Research Institute, Amsterdam, the Netherlands; 3 Department of Gynaecology, OLVG, Amsterdam, the Netherlands; 4 Department of Internal Medicine, OLVG, Amsterdam, the Netherlands; 5 Department of Medical Psychology, Amsterdam UMC, University of Amsterdam, Amsterdam, The Netherlands; 6 Department of Medical Psychology, Amsterdam UMC, Vrije Universiteit Amsterdam, Amsterdam, The Netherlands; 7 Department of Psychiatry, Amsterdam UMC, Vrije Universiteit Amsterdam, Amsterdam, the Netherlands

**Keywords:** Gestational diabetes, Diabetes distress, Adverse pregnancy outcomes

## Abstract

**Background:**

Around 12% of pregnant women develop gestational diabetes mellitus (GDM), which is associated with increased health risks for both mother and child and pre- and postpartum depression. Little is known about the relationship of GDM with diabetes-specific emotional distress (diabetes distress). The aims of this study are to assess the prevalence of diabetes distress in GDM and its association with adverse pregnancy outcomes.

**Methods:**

A prospective cohort study was carried out in an Amsterdam based teaching hospital with an ethnic diverse population. Women diagnosed with GDM completed a set of questionnaires at three time points. Questionnaires consisted of Problem Areas in Diabetes Scale 5 (PAID-5) for diabetes distress (T0-T1), Patient Health Questionnaire 9 (PHQ-9) for depressive symptoms (T0-T2), and questions to assess adverse pregnancy outcomes (T2). Adverse pregnancy outcomes (collected via self-report and if feasible from the medical records) were defined as hypertension, pre-eclampsia, caesarean section, severe perineal tearing, postpartum hemorrhage, postpartum depression, shoulder dystocia, neonatal hospitalization, macrosomia, jaundice, hypoglycemia and other (among which low heart rate, fever, hypoxia). Adverse pregnancy outcomes were dichotomized into none and 1 or more. Additional information was collected from the medical charts. Missing data were imputed via predictive mean matching and a multivariable logistic regression analysis was performed with diabetes distress, depressive symptoms, socioeconomic status, parity and ethnicity as predictors and age, HbA1c, and BMI as covariates.

**Results:**

A total of 100 women were included, mean age 32.5 (4.1), mean BMI 26.7 (4.8), 71% were of non-Dutch ethnic background. Elevated diabetes distress (PAID score ≥ 8) was reported by 36% of the women. Multivariable logistic regression analyses revealed that both high diabetes distress (OR 4.70, *p* = .02) and parity (OR 0.21, *p* = .02) but not antepartum depressive symptoms were related to adverse pregnancy outcomes.

**Conclusions:**

Diabetes distress is likely in women with GDM and our findings suggest an association between both diabetes distress, parity and adverse pregnancy outcomes in women with GDM. This warrants replication and further research into the underlying mechanisms explaining the impact of diabetes distress in GDM and potential interventions to reduce distress.

## Background

Gestational diabetes mellitus (GDM) is present in about 12% of pregnant women [1]. It is not only associated with adverse pregnancy outcomes, among which pre-eclampsia, severe perineal trauma and fetal macrosomia [2–4], but also with an increased maternal risk of developing type 2 diabetes later in life [5, 6].

In addition to these adverse effects, GDM is related to worse psychological outcomes, such as anxiety [7, 8], impaired quality of life [9], and both pre- and postpartum depression [10–12]. Another form of psychological distress in persons with diabetes is diabetes-specific emotional distress, in short diabetes distress [13]. This is defined as negative emotions and worries directly related to the experience of living with and managing diabetes, such as fear for complications and coping problems.

Diabetes distress and depression are two constructs that partly overlap, but they are not one and the same phenomenon [14]. Depression comprises general symptoms such as mood complaints and anhedonia, while diabetes distress specifically relates to the diabetes and its consequences for daily life.

In contrast to diabetes type 1 and diabetes type 2, GDM is a condition that lasts for a short period of time, i.e. during the course of pregnancy. Despite this fact, diabetes distress also occurs in women with GDM. Only a few studies investigated the prevalence of diabetes distress in this population, describing a prevalence rate of up to 40% [10, 15, 16]. Two-third of the women with GDM fear possible consequences for their child, while one-third is also worried about possible congenital malformations, fears that may resemble diabetes distress [7].

Previous studies have been inconclusive about the relation between maternal stress and perinatal complications. Egan et al. (2017) reported no association between antepartum depression, stress or anxiety and pregnancy outcomes such as preterm delivery, delivery type or infant Apgar scores in women with GDM, type 1 diabetes or without diabetes [16], while other studies did report an association between prenatal maternal stress and perinatal complications in pregnant women in general [17, 18].

To date, the possible association between diabetes distress and adverse pregnancy outcomes has not been examined in women with GDM. We aimed to bridge this gap and to determine the extent to which diabetes distress occurs during pregnancy in GDM and whether it is associated with adverse pregnancy outcomes.

## Methods

### Study design

This study represents a prospective cohort of consecutive women who were diagnosed with GDM, based on an oral glucose tolerance test (OGTT) which was performed from the 24th week of pregnancy onwards. Patients with positive results on the OGTT (140 mg/dL (7.8 mmol/L) 2 hours after drinking the glucose solution [19] were referred to the internal medicine department of OLVG, a teaching hospital with an ethnic diverse population in Amsterdam, the Netherlands. The study cohort comprised patients with GDM who visited the small group consultation of the diabetes specialist nurse and medical specialist. Inclusion criteria were being pregnant and having GDM diagnosed < 34 weeks. Exclusion criteria were being under the age of 18 years, having a diagnosis of Type 1 or Type 2 diabetes and being unable to fill in the questionnaires due to insufficient mastery of the Dutch language. Patients were asked to provide written informed consent and to complete a set of questionnaires prior to their first appointment following group consultation, around pregnancy week 28–32 (T0). The interactive group consultation that took place after T0 consisted of a 1-h session with an internist and a diabetes specialist nurse. Patients received information pertaining to gestational diabetes and were given the opportunity to ask questions and share concerns.

After group consultation, approximately 1 month later they were asked to complete the same questionnaires again (T1), on the condition that they were still pregnant. They were contacted once more to fill in questionnaires post-partum, to assess depressive symptoms and adverse pregnancy outcomes (T2), around 4 months post-partum. Diabetes-distress was not measured at T2, since women were recovered from GDM at this point. The study received ethical approval from the medical ethics committee (ACWO-MEC) of OLVG, reference number WO 13–051. According to an a-priori power analysis, 102 women needed to be included to detect a medium difference (Cohen’s *d* 0.50) in adverse pregnancy outcomes, with a power of 0.90 and a significance level of 0.05 [20]. This power calculation is based on a Cohen’s d of 0.50, which means it is powered to detect a difference of 69% of the women with diabetes distress having more adverse pregnancy outcomes than those without diabetes distress.

### Measures

The following information was obtained by means of self-report measures.

### Diabetes distress

In order to assess diabetes distress we used the Dutch version of the 5-item Problem Areas In Diabetes Scale 5 (PAID-5), a validated short form of the PAID-20 [21, 22]. While the PAID has been used in women with GDM before, it has not been validated yet in this population. In patients with diabetes, Cronbach’s alpha of the PAID5 varies from .83 to .86 and its sensitivity and specificity have found to be 94 and 89% respectively [23]. The questionnaire consists of 5 items, scored on a 5-point Likert scale ranging from 0 (not a problem) to 4 (a serious problem). Total scores can range from 0 to 20, with higher scores suggesting greater diabetes- distress. A cutoff of 8 or higher indicates elevated diabetes distress [23]. The PAID-5 was administered at T0 and T1, with a Cronbach’s alpha of 0.81 at T0 and 0.83 at T1.

### Depressive symptoms

In order to assess depressive symptoms pre- and postpartum we used the Patient Health Questionnaire 9 (PHQ-9). Cronbach’s alpha varies from .86 to .89 [24], and its use is validated in pregnant women [25]. The PHQ-9 score ranges from 0 to 27: each of the 9 items is scored from 0 (not at all) to 3 (nearly every day), in which higher scores indicates more depressive symptoms [24]. Research has shown that a cutoff of 12 or higher is suitable to identify elevated depressive symptoms in patients with diabetes in the Netherlands [26]. The PHQ-9 was administered at T0, T1 and T2. Cronbach’s alpha of the PHQ9 was 0.86 at T0, 0.78 at T1 and 0.87 at T2. A score above cutoff at T0, T1 or both was classified as elevated antepartum depressive symptoms. A score above cutoff at T2 was classified as elevated postpartum depressive symptoms.

### Ethnicity and socioeconomic status

Ethnicity was determined based on the country of birth of one’s mother. Country of origin is considered a useful method for defining ethnicity in the Netherlands, since it highly correlates with self-classified ethnicity [27]. Socioeconomic status was estimated (dichotomized as low or average) based on postal area codes of the participants home address. Postal area codes are linked in a national registry to income, population density, mean % of unemployment (excluding students), and mean educational level [28].

### Adverse pregnancy outcomes

Since not every patient delivered in the hospital, we were unable to collect pregnancy and birth outcomes from the medical charts for all patients, except for those who delivered in OLVG. Therefore we decided to collect data from adverse pregnancy outcomes via a self-administered questionnaire (T2). Besides information on postpartum depression, we obtained the following information of the mother and delivery complications: hypertension, pre-eclampsia, caesarean section, (any) perineal tearing, shoulder dystocia, postpartum hemorrhage, other (any other complications that were described by the mother). The following information of the child was obtained: birth weight in grams, hospitalization, macrosomia, jaundice, hypoglycemia, other (among which low heart rate, fever, hypoxia). Terminology was simplified were possible for the self-administered questionnaire. For instance ‘hypertension’ was described as ‘high blood pressure’ and ‘hypoglycemia’ was described as ‘low blood sugar’. To ensure a sufficient number of adverse pregnancy outcomes for analyses, we decided to dichotomize into none and 1 or more. In order to have some information on the validity of the self-reported adverse pregnancy outcomes, these were collected in two ways for women who delivered in OLVG: both self-reported and from the medical charts.

### Medical charts

The following information was extracted from the medical charts:

age, body mass index (BMI), glycated hemoglobin (HbA1c), parity (primiparous or multiparous women), insulin use, history of psychological distress (defined as any history of psychological distress in the medical charts, e.g. depression, anxiety, personality disorder) and having concomitant chronic disease (dichotomized as none or 1 or more). Self-monitoring blood glucose levels (SMBG) were dichotomized into well-controlled or poorly controlled (fasting > 95 mg/dL (> 5.3 mmol/L) and postprandial > 126 mg/dL (> 7 mmol/L)).

### Statistics

We opted for a dichotomization of both depressive symptoms and diabetes distress in order to facilitate interpretability and because in clinical practice a simple screening tool as PAID-5 is often used to discriminate between a deviating and normal level of distress. A score above cutoff at T0, T1 or both was classified as elevated in order to optimize the amount of high PAID5- and PHQ9-scores for analyses. Missing values from the questionnaires were imputed if two or less answers were missing. Data were imputed by multiple imputation with predictive mean matching to avoid negative values, which is known to be valid even in small samples [29, 30]. The total number of 6 imputations was based on the percentage of missing values, since 6% missing data were imputed [31]. All analyses were performed based on the pooled results of these 6 imputations. Since age, diabetes regulation, BMI, SES and ethnicity are known confounders for adverse pregnancy outcomes, these were included in a multivariable regression analysis [32–36]. Other independent variables showing *p*-values < .20 were eligible for multivariable logistic regression analysis as well, which was achieved through the enter method. T-tests and χ^2^-tests were performed to evaluate differences in baseline characteristics. Significance level for baseline variables and multivariable regression analysis was set at *p*-value < .05. Variance Inflation Factors (VIF’s) were calculated to assess multicollinearity. Only variables with VIF’s of less than 4 were included in the multivariable analyses in order to avoid multicollinearity [37]. A Hosmer and Lemeshow goodness-of-fit test was performed to assess the fit of the multivariable logistic regression analysis [38]. All data analyses were performed using SPSS version 21.0 [39].

## Results

**Fig. 1:**
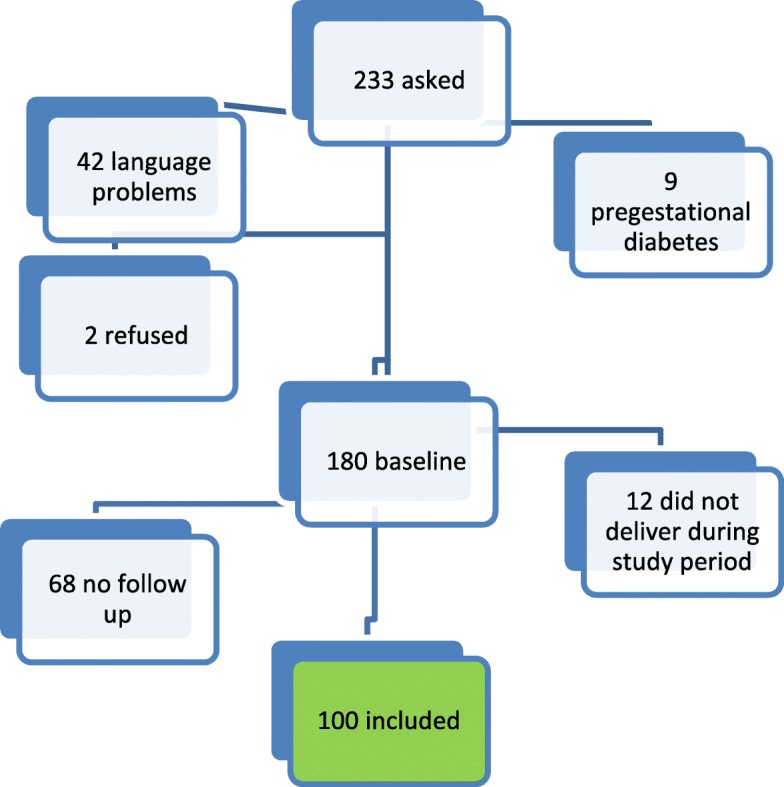
Flowchart of data collection. Legend: 233 women were asked to participate, of which 9 had pregestational diabetes, 2 refused and 42 had language problems. A total of 180 women completed baseline measures, of which 68 did not complete follow-up due to unknown reasons, and 12 did not deliver during the study period. A total of 100 women were included

A total of 233 women were asked to participate, of which 2 refused participation, 9 had been diagnosed with pre-gestational with Type 1 or Type 2 diabetes, and 42 were unable to fill in the questionnaires due to language difficulties. A total of 180 women completed the baseline questionnaire, of which 12 did not deliver in time for the T2 questionnaire, and 68 did not fill in the T2 questionnaire for unknown reasons. A total number of 100 women completed all three questionnaires and were included in the analyses. T0 was completed at a mean pregnancy duration of 27.8 weeks (3.8), T1: mean pregnancy duration of 31.7 weeks (1.3), T2: 4.8 months post-partum (2.6). T2 was completed 5 months post partum (Fig. 1).

No significant differences were found between participants and non-participants with regard to age (*p* = .50), gestational age (*p* = .64), diabetes distress (*p* = .38) or antepartum depressive symptoms (*p* = .42). Mean age was 32.5 (4.1), mean BMI 26.7 (4.8), mean pregnancy duration at baseline 27.8 weeks (3.8) and 71% was of non-Dutch ethnic background (Table 1). Elevated diabetes distress was reported by 23.0% at T0, and 20.0% at T1. A total of 36.0% reported elevated diabetes distress at either T0 or T1, and 7% reported elevated diabetes distress at both time points. Mean diabetes distress declined from 4.8 at T0 to 3.8 at T1 (*p* < .01). A total of 10.0% reported elevated antepartum depressive symptoms (T0 + T1), and 12.0% reported elevated postpartum depressive symptoms (T2). All baseline characteristics are reported in Table 1. Women with diabetes distress more often had a history of psychological distress (*p* < .05), there was no indication that they had poorer glucose regulation than those without diabetes distress: mean HbA1c-levels and insulin use did not differ between women with and without diabetes distress (*p* > .05). The prevalence of depressive symptoms and adverse pregnancy outcomes is shown in Table 2, for women with and without elevated diabetes-distress.

**Table 1:** Baseline characteristics of participants

	Total (*n* = 100) mean ± SD or n (%)	Low diabetes distress (*n* = 64) mean ± SD or n (%)	High diabetes distress (*n* = 36) mean ± SD or n (%)	*p*-value
Characteristics				
Age (years)	32.5 **±** 4.1	32.3 **±** 4.0	32.9 **±** 4.9	0.46
BMI	26.7 **±** 4.8	26.3 **±** 3.8	27.4 **±** 6.2	0.83
HbA1c	33.1 **±** 4.3	32.4 **±** 3.5	34.5 **±** 5.2	0.82
Baseline pregnancy duration in weeks	27.8 **±** 3.8	27.4 **±** 0.5	28.4 **±** 0.5	0.21
Parity				
0	39 (39.0%)	23 (35.9%)	16 (44.4%)	0.41
1 or more	61 (61.0%)	41 (64.1%)	20 (55.6%)	
History of psychological distress				
Yes	46 (46.0%)	24 (37.5%)	22 (61.1%)	0.02
No	54 (54.0%)	40 (62.5%)	14 (38.9%)	
≥ 1 concomitant chronic disease				
Yes	36 (36.0%)	22 (34.4%)	14 (38.9%)	0.67
No	64 (64.0%)	42 (65.6%)	22 (61.1%)	
Insulin use				
Yes	24 (24.0%)	13 (20.3%)	11 (30.6%)	0.25
No	76 (76.0%)	51 (79.7%)	25 (69.4%)	
SES^a^				
Low	43 (43.0%)	29 (45.3%)	14 (38.9%)	0.54
High	57 (57.0%)	35 (54.7%)	22 (61.1%)	
Ethnicity				
Native Dutch	29 (29.0%)	21 (32.8%)	8 (22.2%)	0.36
Moroccan	37 (37.0%)	24 (37.5%)	13 (36.1%)	
Turkish	16 (16.0%)	7 (10.9%)	9 (25.0%)	
Surinamese	7 (7.0%)	4 (6.3%)	3 (8.3%)	
Other	11 (11.0%)	8 (12.5%)	3 (8.3%)	

^a^socioeconomic status

**Table 2:** Depressive symptoms and adverse outcomes of participants and their newborns

	Total (*n* = 100) mean ± SD or n (%)	Low diabetes distress (*n* = 64) mean ± SD or n (%)	High diabetes distress (*n* = 36) mean ± SD or n (%)	*p*-value
Elevated depressive symptoms antepartum (PHQ9 ≥ 12)	10 (10.0%)	2 (0.03%)	8 (22.2%)**	<0.001
Elevated depressive symptoms postpartum (PHQ9 ≥ 12)	12 (12.0%)	3 (0.05%)	9 (25.0%)	<0.001
Birth weight in grams	3273 **±** 659	3390 **±** 508	3065 **±** 832	0.02
Hospitalization neonatology	19 (19.0%)	11 (17.2%)	8 (22.2%)	0.54
Macrosomia	9 (9.0%)	7 (10.9%)	2 (5.6%)	0.37
Jaundice	8 (8.0%)	3 (4.7%)	5 (13.9%)	0.11
Hypoglycemia	6 (6.0%)	3 (4.7%)	3 (8.3%)	0.47
Shoulder dystocia	2 (2.0%)	2 (3.1%)	0 (0.0%)	0.29
Other*	16 (16.0%)	10 (15.6%)	6 (16.7%)	0.37
Caesarean section	28 (28.0%)	16 (25.0%)	12 (33.3%)	0.38
Hypertension	10 (10.0%)	5 (7.8%)	5 (13.9%)	0.37
Pre-eclampsia	1 (1.0%)	0 (0.0%)	1 (2.8%)	0.18
Perineal tearing	23 (23.0%)	16 (25.0%)	7 (19.4%)	0.53
Episiotomy	9 (9.0%)	3 (4.7%)	6 (16.7%)	<0.05
Postpartum hemorrhage	2 (2.0%)	1 (0.02%)	1 (0.03%)	0.68
Adverse pregnancy outcomes				
Yes	71 (71.0%)	40 (62.5%)	31 (86.1%)	<0.01
No	29 (29.0%)	24 (37.5%)	5 (13.9%)	

*among which low heart rate, fever, hypoxia

### Adverse pregnancy outcomes

Factors that were not associated (*p* > .20) with adverse pregnancy outcomes in univariate regression analyses were insulin use, chronic illness, gender of the baby, SMBG and birth weight. Parity and elevated depressive symptoms were associated with adverse pregnancy outcomes in univariate regression analyses, and were therefore included in the multivariate model. Table 3 depicts the multivariate analysis of elevated diabetes distress, elevated depressive symptoms, SES, parity, age, HbA1c, ethnicity, BMI and adverse pregnancy outcomes.

**Table 3:** Multivariable logistic regression analysis of factors associated with adverse pregnancy outcomes

				95% Confidence interval OR’s	
Predictor	β ± SE	*p-*value	OR		
				Lower	Upper
Constant	1.21 **±** 2.49				
Elevated diabetes distress	1.54 ± 0.65	0.02	4.70	1.32	16.75
Age	−0.05 ± 0.07	0.62	0.98	0.85	1.12
SES	−0.14 ± 0.56	0.72	0.82	0.28	2.45
BMI	0.00 ± 0.00	0.56	1.00	1.00	1.00
Ethnicity	0.19 ± 0.16	0.24	1.39	0.89	1.64
HbA1c	0.02 ± 0.01	0.32	1.02	0.99	1.06
Parity	−1.65 ± 0.67	0.02	0.21	0.06	0.73
Elevated depressive symptoms	1.51 **±** 1.16	0.19	4.52	0.47	43.93

A Hosmer and Lemeshow goodness-of-fit test showed a good fit of the multivariable model: χ^2^ = 6.37, *p* = 0.61. Nagelkerke’s R square = 0.32. Both parity (*p* = .02) and elevated diabetes distress (*p* = .02) were significantly related to adverse pregnancy outcomes in a multivariable logistic regression analysis. The odds of adverse pregnancy outcomes were 4.70 times higher for women with elevated diabetes distress during gestation, compared to women without diabetes distress. Parity was a protective factor for adverse pregnancy outcomes: the odds of an adverse pregnancy outcome were 4.76 times higher for primiparous compared to multiparous women. We performed a sensitivity analysis to assess whether the relation between diabetes distress and adverse pregnancy outcomes would still exist if the continuous range of scores was used for diabetes distress instead of dichotomization [40]. Diabetes distress remained related to adverse pregnancy outcomes (*p* < .05). A sensitivity analysis showed that results were similar for the imputated and non-imputated data set.

### Self-report of adverse pregnancy outcomes

To gain some insight into the reliability of the self-reported adverse pregnancy outcomes, we compared the self-reported outcomes with outcomes from the medical charts. For eighty-one women, the presence or absence of adverse pregnancy outcomes were reported in the medical charts. Seventy-one of the 81 (88%) women reported similar adverse pregnancy outcomes as the medical charts. A sensitivity analysis showed a similar association between diabetes-distress and both self-report and medical data in multivariable analyses. Diabetes-distress and self-report data: β (SE) = 1.54 (0.65), *p* = .02, diabetes-distress and medical data: β (SE) = 1.48 (0.69), *p* = .03.

## Discussion

To our best knowledge, this is the first study to report on the relationship between diabetes distress and adverse pregnancy outcomes in women with GDM. Diabetes distress was related to adverse pregnancy outcomes in multivariable analyses, while antepartum depressive symptoms were not. These results corroborate the notion that diabetes distress and depressive symptoms are two different constructs that should be treated as such, also in women with GDM [14].

Given the scarcity of research in this area, a detailed comparison with previous studies is not possible. However, some comparisons can be made. The prevalence of diabetes distress was 36% in our study, which is in line with previous findings in women with GDM [10]. The level of diabetes distress dropped from 4.8 at baseline to 3.8 at follow-up, while Kopec et al. reported no decline in diabetes distress in women with GDM over a period of 9 weeks [15]. Our patients were offered a group consultation after baseline, and this consultation may have contributed to the observed decline in diabetes distress. Sixteen percent of patients lost their diabetes distress in the first month. On the other hand, 13% of the women developed diabetes distress in the same month, perhaps because of the intensification of treatment and measuring of blood glucose values. The 12% prevalence of postpartum depressive symptoms is comparable with other findings in women with GDM, while the 10% prevalence of antepartum depressive symptoms is relatively low in comparison to other studies, reporting percentages ranging from 8.7 to 36% [16, 41].

We did not find an association between glycemic control and adverse pregnancy outcomes, in contrast to Landon et al. [42]. However, contrary to this study we did not perform an OGTT after baseline, and we dichotomized all adverse pregnancy outcomes. Landon et al. looked at separate adverse pregnancy outcomes, of which some were related to glycemic control while others were not. These differences in methodology could explain differences in outcomes. Similar to our findings, the relation between parity and adverse pregnancy outcomes has consistently been found with primiparous women being at increased risk [43, 44]. This calls for specific attention for adverse pregnancy outcomes in women expecting their first child.

We can speculate about the underlying mechanisms explaining the association between diabetes distress and self-reported adverse pregnancy outcomes in our study. First, it could be that diabetes distress similar to the concept of self-rated health is a subjective measure of one’s health status, that has been found to be predictive of objective health and mortality in the general population [45]. Women with GDM experiencing diabetes distress may accurately notice threats to the pregnancy and risk of adverse pregnancy outcomes. As we did not collect data to test this hypothesis, it remains to be confirmed in future studies. A second pathway linking diabetes distress to worse pregnancy outcomes could be via increased cortisol levels. It could be that diabetes distress leads to increased cortisol, which could increase the risk of adverse pregnancy outcomes. It is known, for example, that higher maternal cortisol levels are associated with preterm delivery [46]. Also, maternal anxiety, which is related to elevated cortisol levels, has previously been associated with adverse pregnancy outcomes [47]. Moreover, elevated cortisol levels have been found in women with comorbid depression and anxiety during pregnancy, specifically in women with pregnancy-related emotional distress [48]. However, we did not measure cortisol in our cohort, so we were unable to test this hypothesis.

Our study has some limitations. First, in order to optimize group sizes for analyses, elevated diabetes distress and depressive symptoms at either one time point or both time points were considered the same. However, it is possible that women who have elevated diabetes distress or depressive symptoms at both time points differ from those with elevated diabetes distress at one time point only. Likewise, women with elevated baseline scores could well differ from those with elevated scores at T1. Unfortunately our sample sizes were too small to investigate these potential differences.

Second, there were only 10 women (10%) who reported above threshold antepartum depressive symptoms in our cohort. Possibly the study was not powered to detect an association between antepartum depressive symptoms and adverse pregnancy outcomes in multivariable analyses. However, our data are in line with Egan et al. (2017) who reported no relation between depression and pregnancy outcomes in women with GDM [16].

Third, approximately one third of the patients were lost to follow-up, which could lead to selection bias. It is possible that women who had more severe postpartum depression or who experienced severe adverse pregnancy outcomes did not return the follow-up questionnaire. This could limit the generalizability of our findings. The same accounts for the study population: 70% were of non-Dutch ethnic descent, which is in accordance with the obstetrical population of OLVG. Diabetes distress is known to be common among minorities [49] and replication in other populations is warranted.

Fourth, for practical reasons, we relied on self-reported adverse pregnancy outcomes. For women who delivered in the hospital, self-reported data corresponded in 88% of the cases with the medical charts and the results remained the same when these data were used in a sensitivity analysis. This indicates that the self-reported measures produced results similar to those from the medical charts. Although the outcomes do not give reason to doubt the reliability of the findings, future studies should attempt to collect objective data from the medical charts for all women.

Fifth, while the PAID-5 has previously been used in women with GDM, it has not been specially designed for nor has it been validated in this population. In our sample the PAID5 appeared to be a reliable instrument but we do not have data on its validity. In the group consultation with their diabetes nurse and internist, women with GDM expressed fears about the possible consequences of GDM for their unborn child in particular. They appeared to be most worried about their unborn child, and less about the risks of GDM for their own health. This aspect is not represented in the PAID5. It may be worthwhile to design a GDM-specific questionnaire to measure diabetes-distress in women with GDM, addressing concerns and stress related to the unborn child as well as themselves.

## Conclusions

In conclusion, our results show that diabetes distress is of importance in women with GDM, and that it is related to adverse pregnancy outcomes. As to the clinical implications of our findings, it is important that clinicians are aware and discuss diabetes distress with their patients. A recent review shows that diabetes distress is responsive to treatment [50], and pregnancy does offer a window of opportunities. Group consultation appears a suitable and practical strategy in addition to individual consultation with a nurse or mental health specialist. A multidisciplinary treatment setting, as described in the MySweetheart trial, may be beneficial [51]. Even an approach as simple as monitoring and discussing diabetes distress as part of routine consultations could help improve distress [52] and subsequently improve pregnancy outcomes. Further research should focus on replicating the findings described in this study and investigating opportunities of prevention and management of diabetes distress, with the aim to diminish adverse pregnancy outcomes in women with GDM.

## Data Availability

The dataset analysed during this study are available from the corresponding author on reasonable request.

## Abbreviations

BMI: Body Mass Index
GDM: Gestational Diabetes Mellitus
HbA1c: Glycated hemoglobin
OGTT: Oral Glucose Tolerance TestPAID5: Problem Areas in Diabetes Scale 5
PHQ-9: Patient Health Questionnaire 9
SES: Socioeconomic Status
SMBG: Self-Monitoring Blood Glucose Levels
VIF: Variance Inflation Factor
